# Clinical and endovascular practice in interventional radiology: a contemporary European analysis

**DOI:** 10.1186/s42155-018-0010-8

**Published:** 2018-06-28

**Authors:** Hong Kuan Kok, Thomas Rodt, Fabrizio Fanelli, Mohamad Hamady, Stefan Müller-Hülsbeck, Miquel Casares Santiago, Florian Wolf, Michael J. Lee

**Affiliations:** 1 Interventional Radiology Service, Department of Radiology, Northern Health, Melbourne, Australia; 2 Department of Interventional Radiology, Beaumont Hospital and Royal College of Surgeons in Ireland, Dublin 9, Ireland; 3 Department of Diagnostic and Interventional Radiology, Hannover Medical School, Hannover, Germany; 4 Department of Vascular and Interventional Radiology, Careggi University Hospital, Florence, Italy; 5 Department of Interventional Radiology, Imperial College, London, UK; 6 Department of Diagnostic and Interventional Radiology, Ev.-Luth. Diakonissenanstalt zu Flensburg - Zentrum für Gesundheit und Diakonie, Flensburg, Germany; 7 Department of Interventional Radiology, Clínica Juaneda, Palma de Mallorca, Spain; 8 Department of Cardiovascular and Interventional Radiology, Medical University of Vienna, Vienna, Austria

**Keywords:** Interventional radiology, Clinical practice, Endovascular intervention, Peripheral arterial disease

## Abstract

**Background:**

The purpose of this survey was to determine the current trends in endovascular practice by Interventional Radiologists (IR’s) across Europe and to understand the engagement by Interventional Radiology (IR) with clinical practice. CIRSE European members were invited to participate in an online survey between July 11th, 2016 and August 8th, 2016. A 54 question survey was created to capture a comprehensive overview of IR endovascular practice and clinical engagement.

**Results:**

Four hundred and five valid responses were received (9.9%) from a broad geographic distribution from across Europe. 76% of IR’s practised in centres with more than 400 beds as 60% worked in an academic or university teaching hospital. 36% dedicated 80–100% of their time to IR and 59% dedicated at least 60% of their time to IR. 24/7 IR on-call was available in the hospitals of 73% or respondents. 78% had dedicated IR nursing staff and 67% had nursing support on-call, 55% had inpatient admission privileges and 27% had dedicated IR inpatient beds. 65% of IR’s had admitting rights to day-case beds. 42% ran IR outpatient clinics and 36% performed ward rounds. 81% of respondents performed peripheral arterial disease (PAD) intervention and IR was the main provider of PAD intervention in 67% of centres. Vascular Surgery or Medicine were the main referrers (71%) to IR for PAD intervention. 37% of centres had a hybrid operating theatre and 80% of IR’s had access to this.

**Conclusion:**

IR remains a substantial player in the field of PAD Intervention. The continued evolution of outpatient clinics and clinical practice is key to retention and future expansion in the field of endovascular therapy for PAD.

## Background

Interventional radiology (IR) as a subspecialty of Radiology, has evolved and matured substantially over the past decade. IR is now an essential part of modern medical practice, offering minimally invasive therapies across a wide spectrum of conditions and body systems, both in the acute and elective setting. In Europe, IR gained recognition in 2009 as a distinct specialty within Radiology from the Union of European Medical Specialists (UEMS). Following this, there have been parallel efforts in many European countries to establish IR as a distinct specialty within Radiology, supported by the Cardiovascular and Interventional Radiology Society of Europe (CIRSE) (Lee et al. 2014). Training, assessment and certification of IR specialists have also become increasingly structured with a defined training curriculum and certification in the form of the European Board of Interventional Radiology (EBIR) diploma which has been widely adopted across Europe and also in Australia and New Zealand (Belli et al. 2014; CIRSE 2010; CIRSE 2013).

Over the past decade, there have been increasing demands for IR services due to the expansion of practice in many areas such as the endovascular treatment of acute conditions (eg. ischaemic stroke, trauma, gastrointestinal tract bleeding and complex endovascular aortic aneurysm repair) and elective embolisation (eg. uterine and prostate artery embolisation) and Interventional Oncology (IO) (Tsetis et al. 2016). Nevertheless, it remains unclear how much progress has been made in the clinical practice of IR across Europe over the past decade since the last survey conducted by CIRSE in 2007 (Keeling et al. 2009). Furthermore, the increasing involvement of other specialties such as Vascular Surgery, Cardiology and invasive Angiology in the endovascular treatment of peripheral vascular disease (PVD) has undoubtedly changed the landscape of IR practice. The purpose of this study was to determine the contemporary trends of clinical and endovascular practice by IR across Europe and to understand the gaps in IR specialty development.

## Methods

All active professional CIRSE European members (*n* = 4090) were invited by email in 2016 to participate in an online survey. Data collection was performed between 11th July-8th August 2016. The survey involved completion of a detailed but simple online response form consisting of 54 questions . The form was designed to be user-friendly and featured a simple button-driven interface to facilitate participation by as many interventional radiologists as possible. Broadly, the questions aimed to capture a comprehensive overview of IR practice including the demographic origin of the respondent, IR experience and workload, type of practice and hospital size, availability of IR outpatient clinics, inpatient beds, admitting privileges, availability of 24/7 IR on-call services, availability of IR nurses, technologic staff and trainees, involvement in endovascular treatment of PAD, local infrastructure and access to hybrid operating theatres as well as details regarding funding and reimbursement for IR procedures.

## Results

A total of 405 valid responses were received with a response rate of 9.9%. The geographic distribution of respondents across Europe are listed in Table 1, with a skewed geographic spread towards the United Kingdom (18%), Germany (14%) and Italy (13%). Respondents practicing in all European countries except Bulgaria, Cyprus, Estonia, Finland, Iceland, Liechtenstein, Lithuania, Malta and Slovenia were represented. There was a homogeneous spread of IR clinical experience represented with almost equal proportions of respondents having practiced for 0–10 years (36%), 10–20 years (32%) and more than 20 years (32%) respectively.

**Table 1:** List of countries by response rate

Country of practice	Percent
United Kingdom	18
Germany	14
Italy	13
Spain	7
Greece	6
Turkey	5
Netherlands	4
Switzerland	4
Austria	4
France	3
Belgium	2
Czech Republic	2
Romania	2
Sweden	2
Ireland	2
Norway	2
Russia	2
Portugal	1
Other	8

### IR clinical practice

**Fig. 1:**
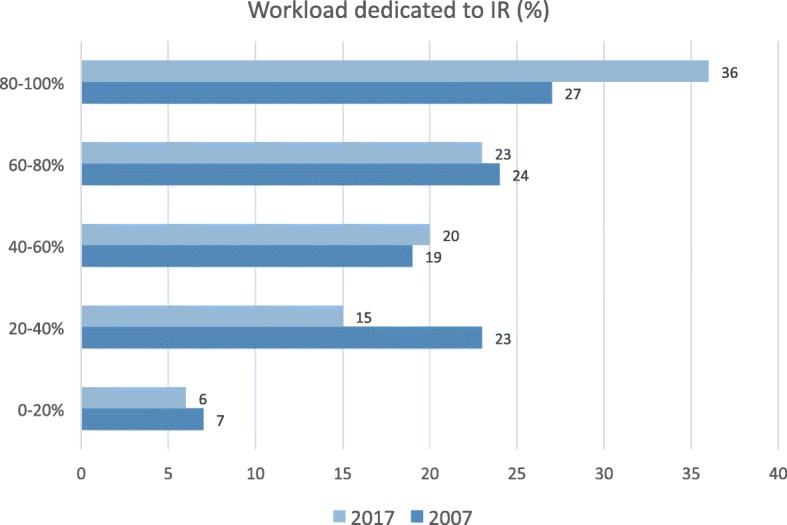
Percentage of respondents’ workload dedicated to IR

Key results from the survey are summarised in Table 2. Most (76%) respondents practiced in centres with more than 400 beds and 42% practiced in large centres with more than 800 beds. Sixty-percent worked in an academic or university teaching hospital and 34% were based in a public general hospital system. A minority of respondents (15%) worked in a private hospital or clinic setting. There were no respondents from private IR facilities. Over one-third (36%) of respondents dedicated 80–100% of their clinical workload to IR and 59% of respondents dedicated at least 60% of their workload to IR (Fig. 1). Almost three-quarters (73%) of respondents practiced in a setting where a 24/7 IR on-call service was provided. Most respondents also indicated that they had dedicated IR nursing staff (78%) in their unit and two-thirds (67%) had nursing support on-call by either a dedicated IR nurse (61%) or a non-IR nurse (6%). Similarly, 61% of respondents had dedicated radiographer/technologist support out of hours.

**Table 2:** Results of survey

Question	Response, % (*n*)
Years of IR practice	
< 10	37 (70)
10–20	30 (56)
> 20	33 (63)
Time dedicated to IR	
0–20%	6 (12)
20–40%	13 (24)
40–60%	16 (30)
60–80%	25 (48)
80–100%	40 (75)
IR day-beds	
Yes	61 (114)
No	39 (73)
Setting of day-bed	
Multispecialty day-ward	36 (41)
Surgical ward	24 (27)
Radiology department	18 (20)
IR ward	12 (14)
Medical ward	9 (10)
Other	1 (2)
IR inpatient admission privileges	
Yes	55 (103)
No	45 (85)
Dedicated IR inpatient beds	
Yes	28 (53)
No	72 (135)
Perform ward rounds	
Yes	36 (65)
No	64 (116)
IR outpatient clinic	
Yes	42 (78)
No	58 (110)
Participate in 24/7 IR on-call	
Yes	73 (137)
No	27 (52)
Dedicated IR nurses	
Yes	78 (145)
No	22 (41)
Nursing support on call	
Yes, dedicated IR nurse	61 (115)
Yes, non-IR nurse	6 (11)
No	33 (63)
Technologist support on-call	
Yes	61 (116)
No	39 (73)
IR trainees at your centre	
Yes	68 (124)
No	32 (59)
Perform endovascular PAD interventions	
Yes	81 (153)
No	19 (36)
Percentage of endovascular PAD treated by IR	
0–20%	10 (15)
20–40%	10 (15)
40–60%	12 (19)
60–80%	15 (22)
80–100%	53 (81)
Hybrid OR in the hospital	
Yes	37 (70)
No	63 (117)
IR access to hybrid OR	
Yes	87 (61)
No	13 (9)

*IR* Interventional radiology, *PAD* peripheral arterial disease, *OR* operating room

**Fig. 2:**
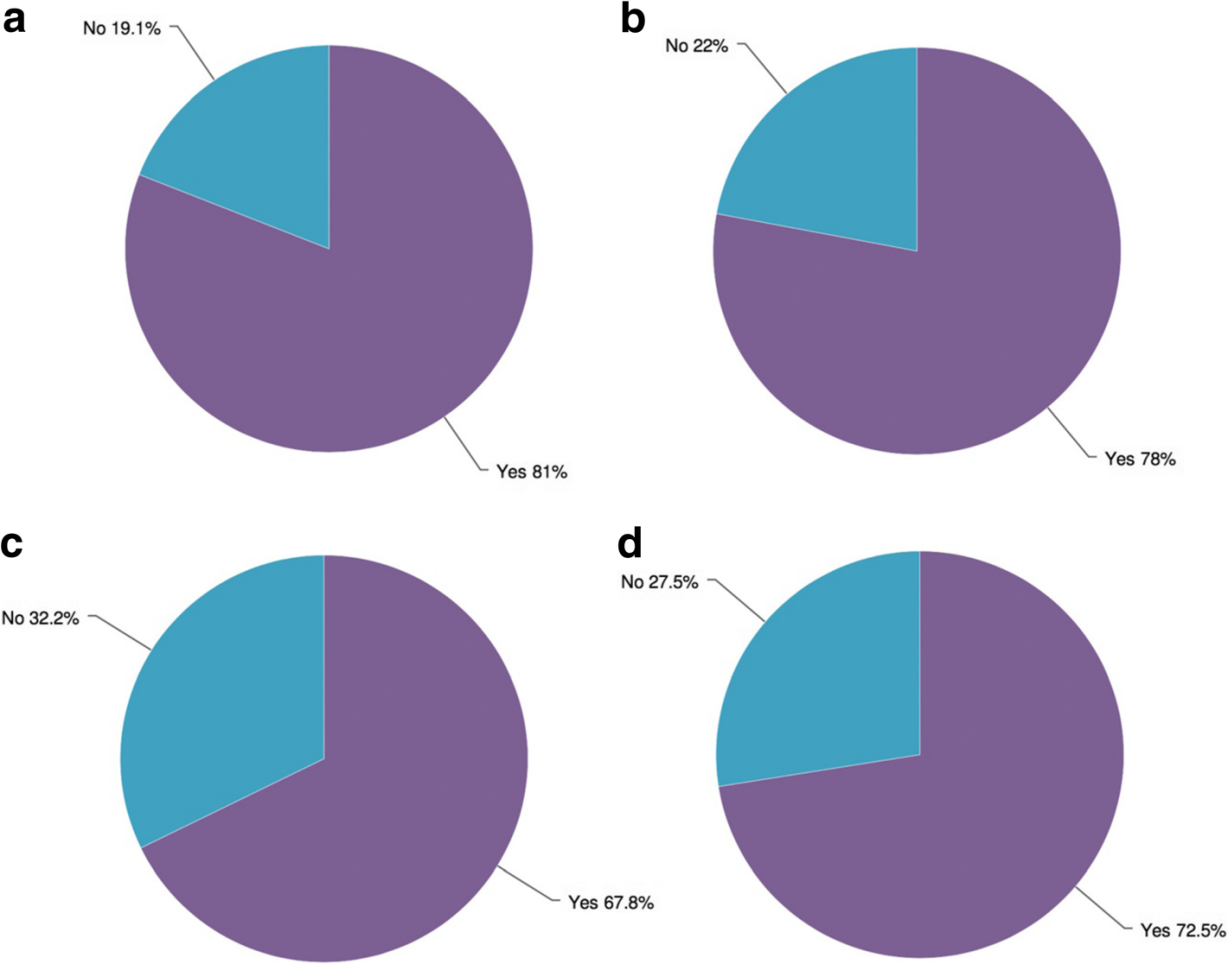
Clinical practice highlights from respondents. **a** Perform peripheral vascular disease interventions. **b** Availability of dedicated IR nursing staff. **c** Have IR trainees. **d** Participate in 24/7 IR on-call

Over one-half of all respondents (55%) had inpatient admission privileges in their centre with just under one-third of respondents (27%) having dedicated IR inpatient beds. More IRs (61%) had admitting privileges to day-case beds. Most of the day-case beds were located in a multispecialty day ward (36%) or surgical ward (24%). A minority of respondents had access to day-case beds within the Radiology department (18%) or a dedicated IR ward (12%). Less than half of all respondents (42%) had IR outpatient clinics and 36% performed ward rounds. Over two-thirds (68%) of respondents had IR trainees in their centre (Fig. 2).

### IR endovascular practice

**Fig. 3:**
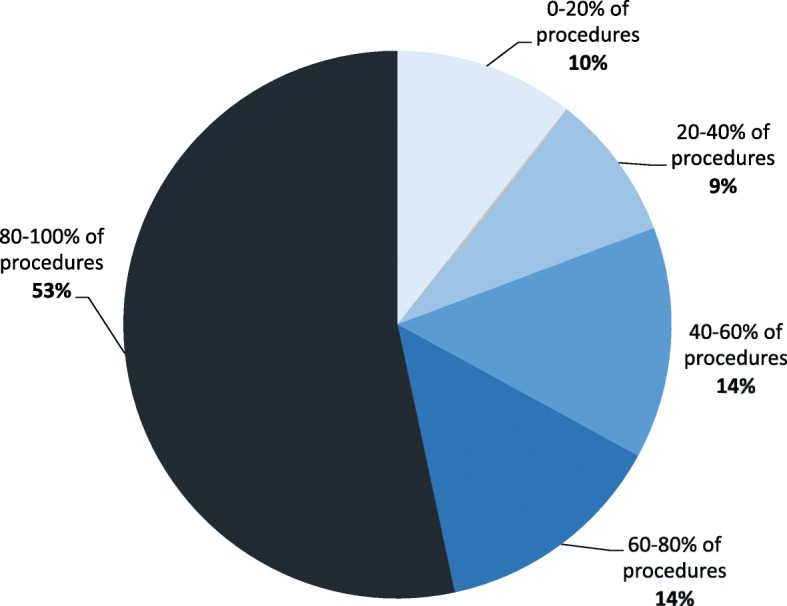
Percentage of peripheral vascular interventions performed by IR at the respondents’ centres

The vast majority (81%) of respondents also performed peripheral arterial interventions and in 67% of centres, IR was the main provider of peripheral arterial interventions covering 60–100% of all peripheral vascular work (Figs. 2a and 3). Apart from IR, vascular surgery (58%), cardiology (19%) and angiology (13%) were the other specialties involved in the endovascular treatment of PAD. Vascular surgery/medicine were the main referrers (71%) to IR for peripheral endovascular procedures. Over one-third of respondents (37%) had a hybrid operating theatre in their hospital and most respondents (87%) had access to this.

## Discussion

IR has come a long way since its humble beginnings in the 1960s and the wide spectrum of minimally invasive image-guided therapies offered by interventional radiologists are now an essential part of modern medicine. However, even the venerable Charles Dotter, father of IR, cautioned in 1968 that radiologists need to assume clinical responsibilities for their practice or risk becoming “high-priced plumbers” and losing out to competing specialties (Rosch et al. 2003). Indeed, trends in the United States at the turn of the twenty-first century have shown an exponential increase in the volume of endovascular peripheral arterial procedures performed by Vascular surgery and Cardiology compared to IR (Levin et al. 2005). Although this is largely due to the move towards an endovascular-first approach to the treatment of PAD and the corresponding increase in volume of endovascular work, the lack of clinical presence by IR, upskilling of competing specialties and diversification of IR practice to other emerging fields such as IO have undoubtedly contributed to this trend (Choke and Sayers 2015).

Nevertheless, the results of our survey show that the majority of European IRs remain clinically active in endovascular peripheral arterial interventions and that in over two-thirds of European centres, IR remains the main provider of endovascular peripheral arterial procedures. Similar to previously observed trends by Levin et al. in the United States, Vascular surgery is the largest competing specialty in Europe with Cardiology and Angiology following behind.

In Europe, IR also remains the leading producer of research in the field of endovascular PAD therapy particularly in the studies related to infrapopliteal and pedal interventions and drug-eluting technology (Asadi et al. 2017). However, it is imperative that interventional radiologists continue efforts to maintain our presence in the endovascular field and produce meaningful academic contributions to advance and lead the way forward in the next generation of endovascular therapies. Current interventional radiologists and IR trainees need to embrace the role of an “IR clinician” who understands the current evidence-base and gaps in knowledge where future research can be directed, is able to clinically assess and manage their patients in a holistic manner to ensure that best clinical care and evidence can be individualized to each specific situation and is technically proficient and safe in performing and delivering minimally-invasive therapies. In addition, the challenging and evolving landscape of endovascular practice requires IRs to be able to work in a multidisciplinary setting and develop partnerships with other referring specialties to ensure that patients continue to benefit from the added value of IR. The setting up of outpatient clinics to take direct patient referral and the attainment of clinical skills during IR training are vital ingredients for the future of our specialty. Teaching IR to medical students in dedicated curricula is also important to educate future referrers and to attract students to IR. Mentoring students, setting up IR electives and fostering research with students in IR are also significant factors in engaging medical students and stimulating an interest in IR.

Since our previous IR clinical practice survey a decade ago, there has been substantial progress in the development of clinical practice within the specialty. More respondents are now dedicating the majority of their clinical commitment to IR, supported by dedicated IR nurses and technologists and the majority also provide a 24/7 IR on-call service. European IR is fortunate to have a large proportion of experienced practitioners, with over two-thirds of respondents having practiced for over 10 years in the field. The proportion of respondents who run IR outpatient clinics has increased from 26% in 2007 to 42% in the current survey, but there remains room for improvement. The number of dedicated IR inpatient beds (17% in 2007 vs 28% currently) remains low. The proportion of inpatient admission privileges has also decreased from 86% in 2007 to 55% in the present survey. However, as many of the procedures we perform can be delivered in an ambulatory setting, respondents had greater access to day-case beds which has increased from 31% in 2007 to 61%. Indeed, the practice of day-case peripheral angioplasty has been established over the past decade and emerging experience with the transradial access technique will facilitate the further expansion of an ambulatory approach to other endovascular therapies (Spiliopoulos et al. 2016; Posham et al. 2016; Huang et al. 2008). Just over one-third of respondents (36%) perform ward rounds in their clinical practice, however this component of practice was not previously surveyed in 2007 to allow accurate comparison. Nevertheless, as interventional radiologists continue to embrace clinical responsibilities, the requirement for presence in both outpatient clinics and wards will undoubtedly follow.

A majority of respondents practice in large tertiary or university teaching hospitals and many have trainees in their unit. The development of IR outpatient and inpatient clinical services should continue to be encouraged to ensure that future generations of IRs have exposure to these essential components of training. Understandably, development of clinical practice varies from institution to institution and progress is commonly hampered by a combination of shortages of manpower, funding and competition for access to limited inpatient beds and support from hospital management or national health service providers. As many respondents are also practicing in larger teaching units, there is a possibility of selection bias in our survey reflecting responses from those who are more active in the endovascular field. Therefore, the results related to endovascular practice may not accurately reflect “real world” practice in all centres. Nevertheless, it does provide a valuable insight into the present state of practice and will serve as a baseline for future comparisons.

## Conclusion

The results from this survey are encouraging in that IR remains a substantial player in the field of endovascular intervention with increasing development of clinical practice models and day-case intervention. The evolution of clinical practice and outpatient clinics is key to the ability to take direct referrals into the future. This will allow IR to maintain a substantial role in the field of endovascular therapy in PAD. Teaching IR to the next generation of doctors is also vitally important, not only to ensure appropriate IR manpower resources into the future, but also to educate future doctors with regard to the utility of IR in treating patients, so that they can refer appropriately.
