# Correction to: Clinical and endovascular practice in interventional radiology: a contemporary European analysis

**DOI:** 10.1186/s42155-018-0023-3

**Published:** 2018-08-21

**Authors:** Hong Kuan Kok, Thomas Rodt, Fabrizio Fanelli, Mohamad Hamady, Stefan Müller-Hülsbeck, Miguel Casares Santiago, Florian Wolf, Michael J. Lee

**Affiliations:** 1 Interventional Radiology Service, Department of Radiology, Northern Health, Melbourne, Australia; 2 Department of Interventional Radiology, Beaumont Hospital and Royal College of Surgeons in Ireland, Dublin 9, Ireland; 3 Department of Diagnostic and Interventional Radiology, Hannover Medical School, Hannover, Germany; 4 Department of Vascular and Interventional Radiology, Careggi University Hospital, Florence, Italy; 5 Department of Interventional Radiology, Imperial College, London, UK; 6 Department of Diagnostic and Interventional Radiology, Ev.-Luth. Diakonissenanstalt zu Flensburg - Zentrum für Gesundheit und Diakonie, Flensburg, Germany; 7 Department of Interventional Radiology, Clínica Juaneda, Palma de Mallorca, Spain; 8 Department of Cardiovascular and Interventional Radiology, Medical University of Vienna, Vienna, Austria

## Correction

Upon publication of this article (Kok et al., 2018), the authors noticed that author ‘Miguel Casares Santiago’ was spelt incorrectly. This has been corrected by means of this correction article.
